# Mitochondrial regulation of local supply of energy in neurons

**DOI:** 10.1016/j.conb.2023.102747

**Published:** 2023-08

**Authors:** Guillermo López-Doménech, Josef T. Kittler

**Affiliations:** Department of Neuroscience, Physiology and Pharmacology, University College London, Gower Street, London WC1E 6BT, UK

## Abstract

Brain computation is metabolically expensive and requires the supply of significant amounts of energy. Mitochondria are highly specialized organelles whose main function is to generate cellular energy. Due to their complex morphologies, neurons are especially dependent on a set of tools necessary to regulate mitochondrial function locally in order to match energy provision with local demands. By regulating mitochondrial transport, neurons control the local availability of mitochondrial mass in response to changes in synaptic activity. Neurons also modulate mitochondrial dynamics locally to adjust metabolic efficiency with energetic demand. Additionally, neurons remove inefficient mitochondria through mitophagy. Neurons coordinate these processes through signalling pathways that couple energetic expenditure with energy availability. When these mechanisms fail, neurons can no longer support brain function giving rise to neuropathological states like metabolic syndromes or neurodegeneration.

## Introduction

Brain information processing requires the constant provision of high levels of energy [1], much of which is consumed to restore the ion movements that underlie neuronal communication and the re-uptake of neurotransmitters [2]. Indeed, organisms have evolved many aspects of their physiology to ensure a constant supply of energy to the brain, and in particular, to the neurons. Pericytes and astrocytes orchestrate a tightly regulated neurovascular coupling to adapt nutrient and oxygen provision in response to increased synaptic activity [3]. Glial provision of lactate or pyruvate to neurons reduces their requirement for metabolic pathways or nutrient stores, not directed at the rapid and efficient production of energy [4,5].

Besides these physiological adaptations [6], the complex architecture of neurons has led to the appearance of a set of cellular mechanisms to closely monitor the metabolic state and to respond to changes in regional/local energy levels to maintain metabolic homoeostasis [7,8].

Mitochondria are home of the oxidative phosphorylation (OXPHOS) machineries that couples ATP synthesis to oxidation of nutrients through the generation of a proton gradient by the mitochondrial electron transport chain. Mitochondrial energetic metabolism governs the pace of neuronal development, and dysfunction leads to neurodevelopmental disease [9]. In addition, mitochondria fulfil other functions like buffering intracellular calcium levels, regulating cell death pathways and hosting numerous biosynthetic pathways [10,11]. Neurons can also obtain their energy from glycolysis, which has a reduced capacity of generating ATP compared to OXPHOS, but is capable to rapidly react to immediate changes in ATP demand, providing a fast response during neuronal stimulation until oxidative metabolism acquire prevalence [6].

Here, we will discuss our current knowledge on the mechanisms that neurons have in place to meet local energetic demands at the mitochondrial level. We will focus on how local metabolic states in neurons control the transport and distribution of mitochondria, how mitochondrial dynamics and mitophagy are controlled spatially, to tune the efficiency of energy generation or to dispose of inefficient mitochondria and what are the signalling pathways that coordinate these processes.

## Transport and distribution of mitochondria to where they are needed

Due to their morphological complexity and large size, neurons have in place mechanisms to control the transport to and distribution of mitochondria at regions of high metabolic demands such as synapses [7,12]. To achieve this, mitochondria associate with KIF5 family kinesins and dynein motors for long range anterograde and retrograde transport along microtubules, respectively [13,14]. The TRAK family of motor adaptor proteins provide directional specificity to mitochondrial transport by preferentially interacting with kinesin (TRAK1) or dynein (TRAK2) [15, 16, 17]. TRAK proteins form complexes with the outer mitochondrial membrane proteins, Miro1 and Miro2, that contain EF hand calcium sensing and GTPase domains [18,19] and regulate the activity of the motor complexes [20,21] with Miro1 playing a more prominent role than Miro2 in this regulation [21,22].

In addition, neuronal mitochondria can regulate their transport and distribution using actin filaments [23] perhaps through mitochondrially localized Myosin19 (Myo19) motor [24,25], which couples to Miro proteins for its OMM recruitment and stabilization [21]. In addition to motor driven transport, a wave of actin polymerization and assembly onto the mitochondrial surface [26] can generate asymmetric comet tails that can drive short and fast mitochondrial movements that were shown to be important to shuffle mitochondrial positioning before mitosis although the relevance of this transport mechanism remains to be proved in neurons [27]. Finally, the actin cytoskeleton can be used as an anchorage for mitochondria thereby securing their position to certain locations like synapses and nodes of Ranvier [28,29] where their function might be needed.

**Figure 1:**
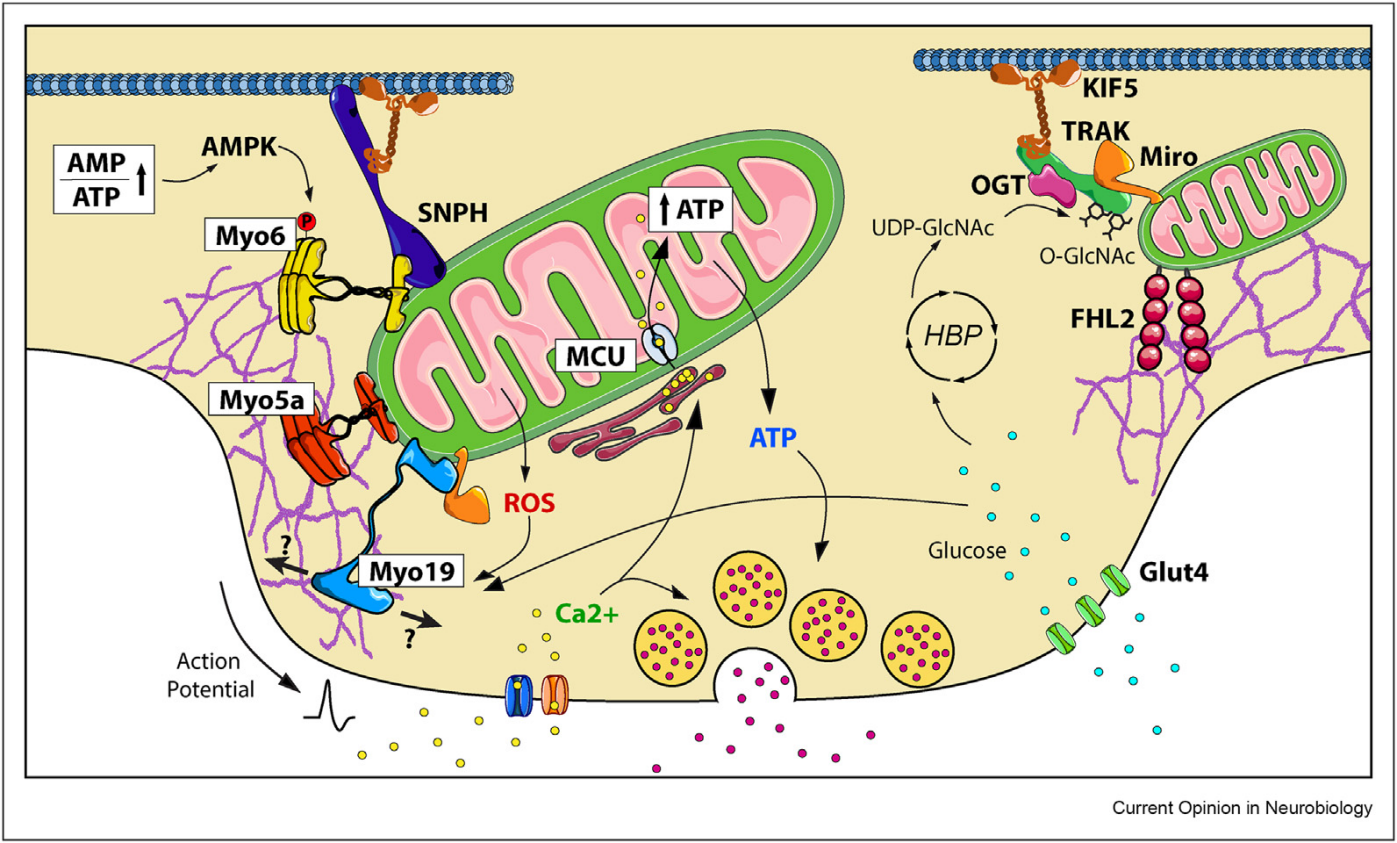
**Synaptic immobilization of mitochondria**, Maintaining synaptic activity is an energetically expensive task. ATP is needed to regenerate the ion gradients responsible for neuronal excitability. Neurons use different mechanisms to ensure the presence of mitochondria near presynaptic sites to ensure that energy provision matches energy expenditure. One mechanism senses the rise in intracellular Ca2+ due to synaptic activation to stop mitochondria via anchorage by Syntaphilin (SNPH). This mechanism is supported by Myosin 6 phosphorylation dependent anchorage of mitochondria to the actin cytoskeleton which is mediated by AMPK and thus, is regulated by the energetic status of the cell. Myosin 5a (Myo5a) and Myosin 19 (Myo19) are other candidates to mediate actin dependent immobilization of mitochondria in synapses, being Myo19 known to be regulated by both ROS production and glucose availability. Mitochondria can also be stopped at the synapse by a mechanisms that senses high glucose levels driven by the increased abundance of glucose transporters. Glucose is used as a substrate to synthetize UDP-GlcNAc by the Hexosamine Biosynthetic Pathway. OGT uses UDP-GlcNAc to catalyze the O-GlcNAcylation of TRAK1, which stops mitochondrial motility and is coupled with the FHL2-dependent anchoring of mitochondria to the actin cytoskeleton.

Mitochondria can adapt their transport and distribution to match local energy requirements through various mechanisms. An activity-dependent rise in intracellular calcium (e.g. at pre- or post-synapses) can arrest motile mitochondria through Miro1 EF-hand-dependent calcium sensing and uncoupling from the transport pathway [30, 31, 32] to increase the presence of mitochondria in the area thus supporting, both energy provision and calcium buffering capacity to regulate synaptic Ca^2+^ homoeostasis [33, 34, 35]. However, synaptic activation-dependent calcium rises can still induce the arrest of motile mitochondria in the absence of Miro1 [22,36], suggesting compensation by Miro2 or the existence of other mechanisms of activity-dependent mitochondrial stopping. For example, syntaphilin (SNPH) allows the immobilization of mitochondria following synaptic activity by binding kinesin motors and microtubules [37] assisted at pre-synapses by Myosin VI (Myo6) and actin filaments [29]. Moreover, the phosphorylation of Myo6 by AMPK [29] implies that synaptic mitochondrial capture is governed by the cellular energetic state as AMPK is activated under energy stress conditions with high AMP/ATP ratios, to increase ATP production and restore energy homoeostasis [38,39] (Figure 1). It is still unclear whether a parallel anchoring mechanism is present in dendrites, where large mitochondria are stationary to support post-synaptic function [40]. Should it exist it might involve a similar Myo6 role, or that of other myosins known to regulate mitochondrial motility or anchorage like Myosin V (Myo5) [28] or Myo19, which in turn is known to respond to glucose starvation and to levels of ROS [41,42] (Figure 1).

Nutrient levels can also influence CNS mitochondrial dynamics. Increased synaptic activity can induce a rise of intracellular glucose by stimulating the membrane accumulation of glucose transporters [43]. Glucose can influence the machinery of mitochondrial transport and regulate mitochondrial motility and distribution through O-linked N-acetylglucosaminyltransferase (OGT), an integral component of the mitochondrial transport machinery [44]. OGT activity responds to local concentrations of UDP-GlcNAc, a high energy donor substrate produced by the hexosamine biosynthetic pathway and thus is a readout of free glucose availability [45]. OGT catalyzes the O-GlcNAcylation of TRAK1 stopping mitochondrial motility [46] through FHL2-dependent anchoring of mitochondria to the F-actin cytoskeleton [47] mediating the accumulation of synaptic mitochondria for an efficient glucose utilization (Figure 1).

Interestingly, enhancing mitochondrial trafficking can also overcome axonal energy crisis and protect against axonal degeneration [48,49]. One mechanism recently identified to enhance axonal motility and protect against injury induced damage involves the local activation of the AKT effector PAK5, which induces the remobilization of stationary damaged mitochondria in axons through phosphorylation of SNPH [50]. This remobilization may help to provide new mitochondria to nerve terminals requiring energy to restore axonal homoeostasis as well as to recycle damaged mitochondria by delivering them to the somatodendritic compartment where they can enter the autophagy pathway [51].

## Mitochondrial remodelling in response to energetic balance

Mitochondrial morphology undergoes remodelling through the antagonistic processes of mitochondrial fusion and fission [52]. Mitochondrial fusion is mediated by the concerted action of three large GTPases, Mitofusin 1 and 2 (Mfn1 and Mfn2) located on the outer mitochondrial membrane (OMM) and OPA1 located on the inner mitochondrial membranes (IMM). Mutations in Mfn2 and Opa1 lead to Charcot-Marie-Tooth 2A [53] and Dominant Optic Atrophy [54,55], respectively, highlighting the importance of fusion processes for neuronal function [53, 54, 55]. Mitochondrial fission depends on the activity of Drp1, another large GTPase critical for neuronal development [56,57]. Drp1 is recruited to the OMM from the cytoplasm by its mitochondrial receptors Fis1, MFF, MID49, or MID51, and assembles in a ring structure surrounding mitochondria, that constricts and induces the fission of both mitochondrial membranes assisted by an ER tubule and the local polymerization of actin [58, 59, 60].

Generally, longer and more interconnected (fused) mitochondria correlate with high respiration efficiency [61] and protection against mitophagy [62]. In contrast, mitochondrial fission helps cells to activate apoptotic programs and increases the mitophagic flow [63]. In neurons, a highly interconnected network is typically seen in the somas and dendritic compartment [64], which may facilitate responding to high energy demands and Ca^2+^ buffering requirements in regions with high density of synapses [40,65]. Mitochondria in axons are shorter, perhaps to facilitate extended transport distances to reach presynaptic sites [66]. Presynaptic capture in terminal boutons of these shorter mitochondria ensures enough local ATP generation and accurate Ca^2+^ buffering capacity required to support synaptic transmission on these presynaptic sites [33,34,67]. Axonal mitochondrial size, dictated in part by Drp1 and MFF, are critical factors in regulating the Ca^2+^ buffering capacity of the organelle and synaptic communication as bigger mitochondria with higher buffering capacity might keep local Ca^2+^ concentration below the threshold required to allow for neurotransmitter release [66].

**Figure 2:**
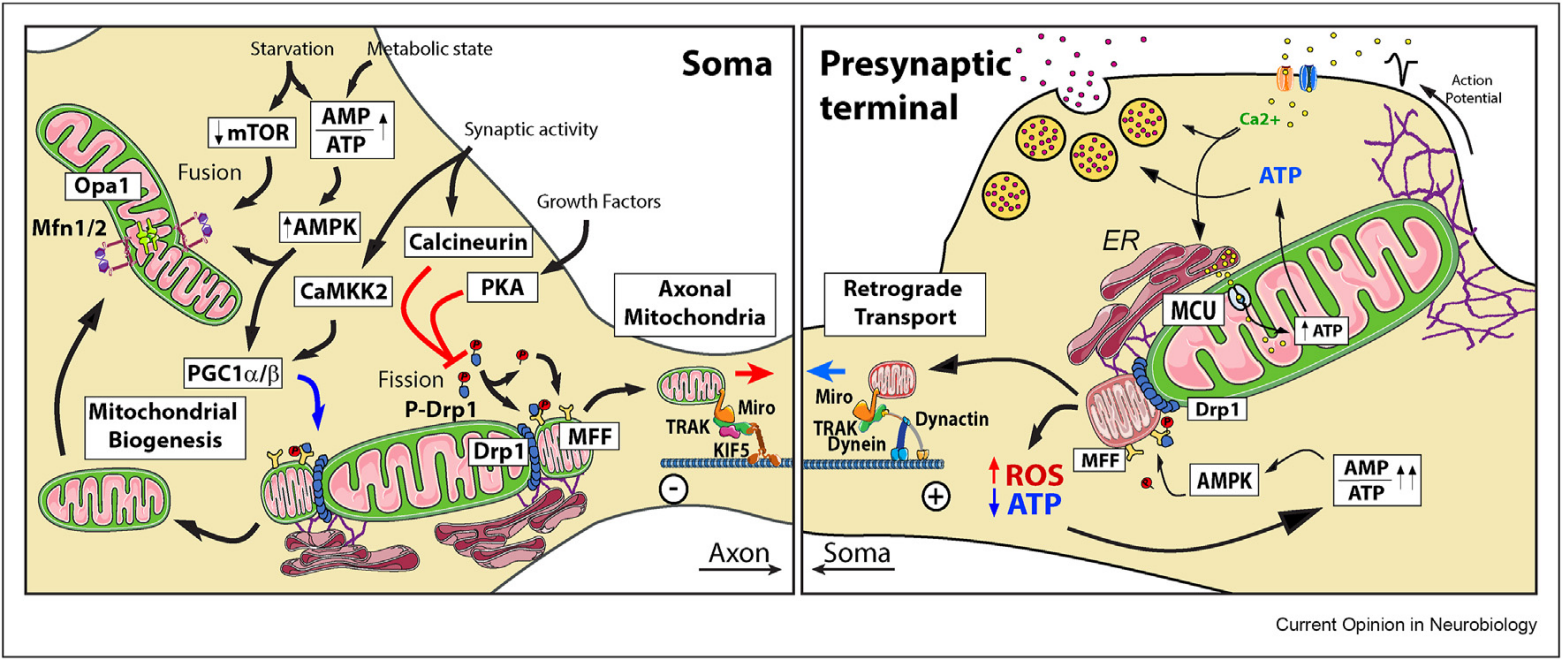
**Mitochondrial dynamics, biogenesis and mitophagy**, Neurons regulate mitochondrial remodelling locally in response to the cellular context. Mild energetic stress favours mitochondrial fusion through the AMPK or mTOR pathways. Growth Factors and synaptic activity, through activation of PKA and Calcineurin respectively, may also stimulate mitochondrial elongation by inhibiting the translocation of Drp1 to the mitochondrial membrane and, thus, inhibiting fission. In contrast, Drp1 and MFF activity are critical to ensure that short mitochondria is produced to enter in the axon and populate presynaptic terminals. In addition, similar signalling pathways, like AMPK or CaMKK2, activated by the energetic state or synaptic communication respectively, can also stimulate mitochondrial biogenesis through activation of PCG1. Continuous synaptic activity giving rise to elevated levels of ROS and energy depletion in axons can activate the fission of defective mitochondria though the strong activation of AMPK, which mediates fission instead of fusion by phosphorylation of MFF and recruitment of Drp1 to the mitochondrial membrane. This process help removed dysfunctional mitochondria by coupling this fission to the retrograde mitochondrial transport machinery to deliver these defective mitochondria to the soma where it will fuse to lysosomes and be degraded.

Studies in non-neuronal cells established that under mild energetic stress like starvation conditions, the mitochondrial network elongates and gains complexity through the induction of mitochondrial fusion in response to activation of the AMPK or inhibition of the mTOR pathways, both protective pathways activated under mild metabolic stress conditions [62,68]. Such increase in mitochondrial fusion is important to sustain cell viability during starvation induced autophagy [69]. In neurons, the AMPK, the PKA/AKAP1 and Calcineurin signalling pathways, activated in response to increased levels of AMP and ADP, extracellular growth signals or synaptic activity, respectively, can also stimulate the elongation of the mitochondrial network by controlling the translocation of Drp1 to the mitochondrial membrane [70,71] (Figure 2). By inhibiting mitochondrial fission, PKA/AKAP1 favours the efficiency of mitochondrial respiration to support energy consumption during neuronal morphogenesis or to protect against ischaemia and cell death after neuronal injury or excitotoxicity [72,73].

## Ca^2+^ and energy production

Activity-dependent cytoplasmic Ca^2+^ rises trigger Ca^2+^ release from the ER which can be taken up by local mitochondria though the mitochondrial Ca^2+^ uniporter (MCU), a Ca^2+^ selective ion channel that opens upon high concentrations of cytosolic Ca^2+^ [74,75]. This mitochondrial Ca^2+^ uptake generally requires the close apposition of ER and mitochondrial membranes at the ER-mitochondrial contact sites (ERMCS) where high concentrations of cytoplasmic Ca^2+^ are achieved [76]. However, recent work has challenged the view that in neurons the close apposition of the ER is required for mitochondrial Ca^2+^ uptake [77]. Presynaptic mitochondria shows a low threshold for Ca^2+^ uptake conferred by the presence of MICU3, a neuronal specific regulator of the MCU complex, which sensitizes these mitochondria to the cytoplasmic Ca^2+^ fluctuations associated with synaptic activity [77].

The uptake of Ca^2+^ by the MCU acts as a feedforward mechanism that boosts mitochondrial metabolism by activating the TCA cycle and the respiratory chain, increasing ATP production in different systems [78, 79, 80]. It is worth noting that this point remains controversial in neurons, where recent studies have shown that activity dependent Ca^2+^ entry might boost synaptic energy homoeostasis by an MCU-independent mechanism [81,82], or that MCU itself, is not required to keep synaptic metabolic homoeostasis [83]. A possible explanation is that an intermembrane space elevation of Ca^2+^ can stimulate the malate-aspartate shuttle and increase pyruvate availability for the TCA cycle contributing to the increase in activity-dependent energy generation [81,82].

Mitochondrial Ca^2+^ uptake can, reciprocally, shape the dynamics of cytosolic Ca^2+^ concentration and thus regulate local Ca^2+^ signalling influencing synaptic transmission or excitotoxicity and metabolism [34,35,77,82,84, 85, 86]. Neurons might control the efficiency of energy generation and Ca^2+^ buffering and thus influence synaptic transmission [76] by controlling the expression levels of MCU, MICU3 or other MCU regulatory subunits [86], or by regulating the amount and functionality of the ERMCS. This can be done by modulating the expression levels and activity of Ca^2+^ regulatory proteins like IP3R/GRP75/VDAC1 [87,88], the ER-mitochondrial tether proteins like VAPB/PTPIP51 [89,90] or the ER localization of Mfn2 [91].

## Mitochondrial biogenesis

The energetic balance of the cell is a strong driving force regulating the generation of *de novo* mitochondria [87]. Mitochondrial biogenesis is a self-renewing mechanism that requires pre-existing mitochondria [88] and can occur locally in any compartment of the cell [89,90]. This is extremely important in neurons as distal axonal mitochondria that need to be replaced would require many days in reaching their final position if they had to travel from the soma. There needs to be coordination between nuclear and mitochondrial processes to ensure the formation of new mitochondria [91]. First, there should be coordinated mtDNA replication as well as nuclear and mtDNA transcription and translation to produce the mitochondrial components required for the new mitochondrial mass [92]. In addition, the coordinated synthesis and import of mitochondrial components encoded by the nucleus is critical to ensure the stoichiometry and functionality of the mitochondrial components [93,94]. Peroxisome proliferator-activated receptor-γ coactivator-1alpha and -beta (PGC-1α and PGC-1β) are master regulators of mitochondrial biogenesis [95,96] regulating mitochondrial density in axons [97]. Both, PGC-1α and PGC-1β, mediate the activation of the mitochondrial transcription factor TFAM [98] and respond to several signalling pathways, like AMPK, LKB1 or CaMKK2, in turn activated by the energetic balance [99,100] or Ca^2+^ induced by NMDA receptor activation [101]. Under energy stress conditions, AMPK phosphorylates and activates PGC-1α [102] and Sirt1, which in turn deacetylates PGC-1α reinforcing its activation [103] (Figure 2). Activated PGC-1α stimulates nuclear transcription of TFAM through the upregulation of NRF1/2 which in turn activates the expression of mitochondrial genes [104]. Mitochondrial biogenesis driven by PGC-1α activation and the switch to OXPHOS metabolism has been shown to be critical during motor neuron development [105] and are associated with protection from motor neuron loss in ALS models [106,107]. PGC-1α deficiency was also associated with GABAergic interneuron function [108] and with loss of dopaminergic neurons in the substantia nigra [109].

## Mitophagy and energetic failure

Mitophagy is especially important in neurons, cells that do not divide and that would accumulate, during their lifetime, damaged and inefficient mitochondria [8]. The most common and better described mitophagy mechanism is the damage-induced mitophagy in which PINK1 and Parkin are central players responsible for the identification, isolation and degradation of defective mitochondria [110]. In addition to mitochondrial damage as a trigger, the cell's energetic balance is a key determinant that regulates mitochondrial quality and quantity through the regulation of the mitophagic flow. Inhibition of mTOR under starvation conditions removes the break on ULK1 activation, which leads to beclin1 phosphorylation and autophagosome formation and facilitates mitophagy [87,111]. Likewise, under severe metabolic stress, AMPK activation was shown to stimulate mitophagy [112] at least in part by a similar activation of ULK1 [113]. In this case, the strong activation of AMPK induces fission rather than fusion by phosphorylating MFF and stimulating the activation of Drp1 on the OMM, allowing for the controlled mitophagy of small fragments of defective mitochondria which can be engulfed by an autophagosome [114] (Figure 2). In an analogous mechanism, PINK1 phosphorylation also induces mitochondrial fission allowing the segregation of damaged mitochondria to undergo mitophagy [115]. Under repeated synaptic stimulation, a presynaptic mitochondria is subject of significant energetic pressure and have its OXPHOS system strained. The increased respiration rate is accompanied by an enhanced generation of radical oxygen species (ROS), which might themselves act as signals to locally induce the recycling of parts of the mitochondrial compartment by mitophagy [116] (Figure 2).

## Regulation of mitochondria substructure

Mitochondrial internal architecture can vary enormously both within and between cell types and can reflect the metabolic state of that mitochondria and of the cell more broadly [117, 118, 119]. In neurons, synaptic mitochondria exhibit higher cristae density than those in other cellular regions [120], suggesting that cristae architecture is dynamic and can be remodelled to adapt to local energetic and Ca^2+^ buffering demands [119,121]. This gives rise to a complex synapse/mitochondrial crosstalk by which synaptic activity shapes mitochondrial ultrastructure [122] and thus the functionality of local mitochondria while, reciprocally, these mitochondria critically regulate synaptic performance and homoeostasis [7,33,65,123].

The components of the fusion and fission machineries control cristae structure by influencing inner membrane dynamics directly (via Opa1) or indirectly (Mfn1/2 and Drp1) by regulating the balance between fusion and fission [124]. In addition, Opa1 accomplishes a role in shaping cristae structure and in controlling the release of cytochrome C during apoptosis that is independent of mitochondrial fusion [125] and which may have additional consequences for synaptic transmission [126]. Opa1 function requires, at least in part, the presence of another cristae membrane remodelling player, the F_1_F_0_-ATP Synthase [127], which forms dimers that induce membrane curvature and are important for the stabilization of mitochondrial cristae [128,129] implying that metabolically active mitochondria ensures its own cristae stability.

**Figure 3:**
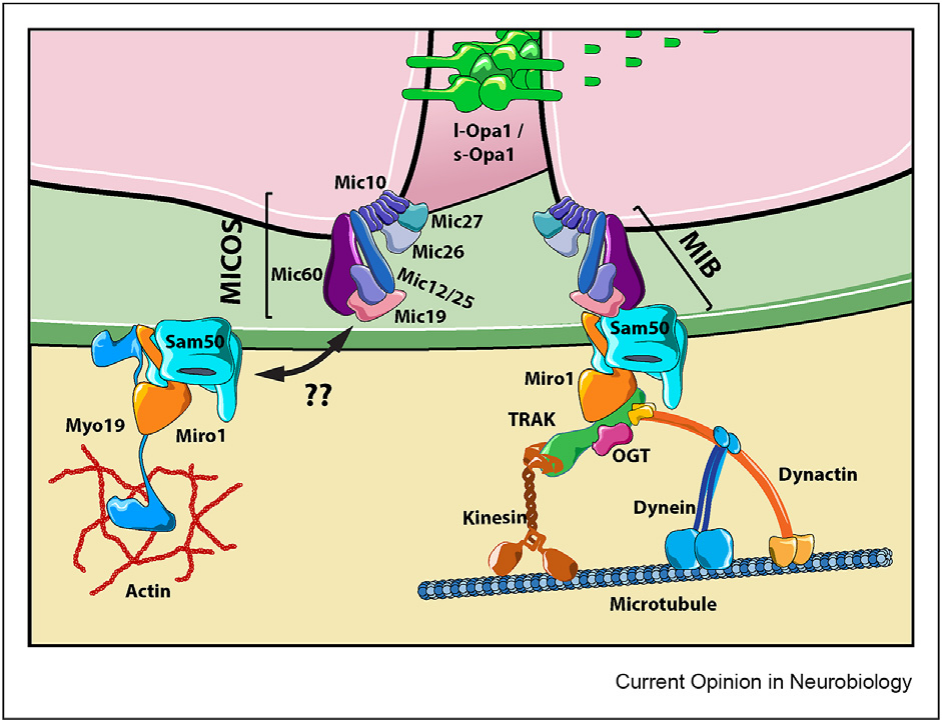
**Regulation of cristae structure by the transport machinery**, Mitochondrial ultrastructure is linked to mitochondrial function. Opa1 and the MICOS complexes are two critical regulators of mitochondrial cristae morphology and thus can potentially control mitochondrial energy production. It has recently been identified a molecular bridge between the MICOS complex and the mitochondrial transport machinery that couples both mitochondrial membranes to the transport pathway. This bridge may act as a sensor of intramitochondrial oxidation and thus influence the transport of the organelle. Likewise, the signalling pathways governing mitochondrial trafficking might impact mitochondrial ultrastructure and ultimately energy production.

The Mitochondrial Contact Site and Cristae Organising System (MICOS) is another critical regulator of the IMM substructure [130]. MICOS is a large hetero-oligomeric complex composed of two core subcomplexes, Mic60 and Mic10, which accomplishes important roles in the formation and stabilization of tubular or lamellar cristae, respectively [131,132]. MICOS binds the mitochondrial intermembrane space bridging complex (MIB) or Sam complex to form the MICOS-MIB, which attaches both mitochondrial membranes onto the same macromolecular complex [133] (Figure 3). A number of mutations in MICOS components have been identified in patients with different neurodegenerative diseases [134]. Mutations in Mic60 (mitofilin) has been identified in Alzheimers's disease [135] and Parkinson's disease [136], while mutations in Mic14 (CHCHD10) have also been reported in Alzheimers's disease [137] and Charcot-Marie-Tooth [138], highlighting the critical dependence of neurons of an appropriate IMM ultrastructure.

Super-resolution imaging has allowed the visualization and characterization of the dynamic nature of individual cristae [139] highlighting the idea that cristae are isolated and functionally independent structures that can display different membrane potentials [140]. This advocates for the existence of regulatory mechanisms of energy production involving the participation of a small proportion, or even only one cristae to boost a specific cellular process locally. This idea is supported by the fact that the mitochondrial cristae and the MICOS complexes controlling their structure follow particular, non-random distributions throughout the mitochondria [141]. Moreover, we have known for over a decade that cristae junctions are distributed asymmetrically in presynaptic mitochondria with the mitochondrial side displaying high density of cristae junctions facing the active region of the presynaptic membrane [120]. The recent identification of a molecular bridge between the mitochondrial transport machinery and the MICOS complex [142] that may additionally serve as a transducing machinery of intramitochondrial oxidation [143] and in which metaxins might be involved [144] suggests that the mechanism of cristae junction distribution at such small spatial scale might be regulated by similar cues and signalling pathways as the mitochondrial transport throughout the neuron (Figure 3). The fact that Myo19 controls cristae architecture and energy production [145] and ER-mitochondria association and mitochondrial fission [146] reinforces the vision of an integrated mitochondrial transport and dynamics with the efficiency in energy generation of mitochondrial cristae.

## Conclusion

How neurons regulate the local supply of energy in the regions where it is needed is critical to sustain neuronal homoeostasis and function. We have discussed here how this entails the integration of numerous signalling pathways that control the molecular mechanisms governing mitochondrial trafficking and distribution, mitochondrial dynamics, as well as biogenesis and turnover. We argue that a more profound knowledge and understanding of the mechanisms underpinning the metabolic regulations of mitochondrial function, including those governing mitochondrial ultrastructure and bioenergetic function, will provide novel targets for therapeutic intervention to treat a vast range of pathologies in which mitochondrial function is compromised, from metabolic syndromes, neurodegenerative diseases, neurodevelopmental disorders or even pathologies associated with ageing and cancer.

## Author contributions

GL-D and JTK wrote the paper.

## Declaration of competing interest

The authors declare that they have no known competing financial interests or personal relationships that could have appeared to influence the work reported in this paper.

## Data Availability

No data was used for the research described in the article.
